# Variability of toe pressures during haemodialysis: comparison of people with and without diabetes; a pilot study

**DOI:** 10.1186/s13047-023-00642-y

**Published:** 2023-07-10

**Authors:** Rachel Carle, Peta Tehan, Sarah Stewart, David Semple, Andrew Pilmore, Matthew R. Carroll

**Affiliations:** 1 Community and Long-Term Conditions Directorate, Te Toka Tumai, Auckland, New Zealand; 2 Department of Surgery, School of Clinical Sciences, Faculty of Medicine, Nursing and Allied Health, Monash University, Clayton, VIC Australia; 3 Department of Podiatry, School of Clinical Sciences, Faculty of Health and Environmental Sciences, Auckland University of Technology, Private Bag 92 006, Auckland, 1142 New Zealand; 4 Active Living and Rehabilitation, Aotearoa New Zealand, Health and Rehabilitation Research Institute, School of Clinical Sciences, Auckland University of Technology, Auckland, New Zealand; 5 Department of Renal Medicine, Te Toka Tumai, Auckland, New Zealand; 6 School of Medicine, University of Auckland, Auckland, New Zealand

**Keywords:** End-stage renal disease, Diabetes mellitus, Haemodialysis, Toe systolic blood pressure, Toe-brachial index

## Abstract

**Background:**

Diabetes, end stage renal disease (ESRD), and peripheral arterial disease (PAD) are associated with a higher risk of diabetes-related lower limb amputation. Timely identification of PAD with toe systolic blood pressure (TSBP) and toe-brachial pressure index (TBPI) is critical in order to implement foot protection strategies to prevent foot complications in people with ESRD. There is limited evidence describing the effect of haemodialysis on TSBP and TBPI. This study aimed to determine the variability of TSBP and TBPI during haemodialysis in people with ESRD, and to determine whether any observed variability differed between people with and without diabetes.

**Methods:**

TSBP and TBPI were taken before dialysis (T1), one hour into dialysis (T2) and in the last 15 min of dialysis (T3) during a single dialysis session. Linear mixed effects models were undertaken to determine the variability in TSBP and TBPI across the three time points and to determine whether this variability differed between people with and without diabetes.

**Results:**

Thirty participants were recruited, including 17 (57%) with diabetes and 13 (43%) with no diabetes. A significant overall reduction in TSBP was observed across all participants (*P* < 0.001). There was a significant reduction in TSBP between T1 and T2 (*P* < 0.001) and between T1 and T3 (*P* < 0.001). There was no significant overall change in TBPI over time (*P* = 0.62). There was no significant overall difference in TSBP between people with diabetes and people with no diabetes (mean difference [95% CI]: -9.28 [-40.20, 21.64], *P* = 0.54). There was no significant overall difference in TBPI between people with diabetes and people with no diabetes (mean difference [95% CI]: -0.01 [-0.17, 03.16], *P* = 0.91).

**Conclusion:**

TSBP and TBPI are an essential part of vascular assessment of the lower limb. TBPI remained stable and TSBP significantly reduced during dialysis. Given the frequency and duration of dialysis, clinicians taking toe pressures to screen for PAD should be aware of this reduction and consider how this may have an impact on wound healing capacity and the development of foot related complications.

## Background

Diabetes is the most common cause of kidney failure, accounting for 47% of all new end stage renal disease (ESRD) cases in New Zealand in 2019 [1].The progression of microvascular kidney damage to ESRD in diabetes is associated with increased prevalence of peripheral neuropathy and peripheral arterial disease (PAD), which is subsequently associated with an increased risk for diabetes-related lower limb amputations [2]. Foot complications such as ulceration, infection, gangrene and amputation are two-fold more prevalent in persons with ESRD compared to non-nephrotic persons with diabetes [3].

ESRD can lead to uremic neuropathy through an accumulation of dialysable neurotoxins during haemodialysis [4]. Uremic neuropathy is a distal sensorimotor polyneuropathy that leads to a loss of protective sensation in both people with and without diabetes undergoing dialysis [5]. Both uremic and diabetic neuropathy can result in a disruption of the arterio-venous shunting process, leading to capillary circulation being bypassed and vital nutritional and gas exchanges being impaired [6]. This is associated with increased fissuring and infection rates in these populations [6]. There is a strong association between ESRD, loss of protective sensation (LOPS) and diabetes-related lower limb amputation, with a 6.5- to tenfold higher likelihood than in the general diabetes population [3, 7]. Additionally, lower limb amputation is prevalent in ESRD and diabetes populations, regardless of the presence of both conditions. Both ESRD and lower limb amputation lead to a reduction in quality of life and an increased risk of premature mortality [8]. Foot ulceration and amputation requiring vascular intervention is an expensive burden for taxpayers, with median costs for treatment estimated at $30 K NZD per wound [9].

Measurement and monitoring of peripheral blood flow using non-invasive vascular assessments (Doppler waveform analysis, ankle-brachial index, toe-brachial pressure index (TBPI), toe systolic blood pressure (TSBP) can provide information on presence and progression of PAD and expedite triage to vascular services, which may reduce the risk of lower limb amputation [10, 11]. TSBP can be measured chairside using a suitable hand-held Doppler, which provides a valuable measure of peripheral blood perfusion [12]. TSBP < 30 mmHg (non-pathologic TSBP > 60 mmHg) [11] is associated with a relative risk of 3.25 for amputation and non-healing [13]. The TBPI, which compares TSBP to brachial systolic blood pressure, is another important indicator for PAD, with results of ≥ 0.75 making the diagnosis of PAD less likely [14].

There is limited research describing peripheral vascular assessment in people with concomitant diabetes and ESRD during dialysis. Kay et al. [15] reported TSBP values reduced from mid to post-dialysis in persons with diabetes, but not in persons with no diabetes. There have been a small number of other studies related to peripheral blood flow during dialysis, but only one related to TSBP variability [8, 16–19]. Tsuyuki et al. [11] compared the ankle brachial index (ABI) to TBPI in people with ESRD and found that TSBI showed a lower level of specificity than the ankle-brachial pressure index, attributing this finding to extensive medial arterial calcification, which is frequently present in ESRD [11]. The sensitivity and overall diagnostic accuracy of the ABI in detecting 50% or greater arterial stenosis in individuals with chronic kidney disease have been shown to be 43% and 67%, respectively [20]. In contrast, the sensitivity and overall diagnostic accuracy for abnormal TBPI in detecting 50% or greater arterial stenosis were 77% and 72% in individuals with chronic kidney disease. For those with inconclusive ABIs, sensitivity and diagnostic accuracy of TBPI were 75% and 69% [20]. The authors of this study concluded that TBPI should ideally be used to complement or supplement ABI. Additionally, The American College of Cardiology/American Heart Association recommends using TBPI in evaluating patients with falsely elevated ABI, specifically in people with diabetes and those with chronic kidney disease because of the higher prevalence of medial arterial calcification of the tibial arteries [21].

The primary aim of this study was to determine the variability of TSBP and TBPI during haemodialysis in people with ESRD. The secondary objectives were to determine whether observed variability in TSBP and TBPI was different between participants with and without diabetes.

## Methods

This cross-sectional pilot study was conducted between October and December 2022. Potential participants were recruited from two community dialysis clinics in Auckland, New Zealand (Kererū Dialysis Centre and Carrington Dialysis Centre).

### Inclusion criteria

Participants were included if they had ESRD, were on haemodialysis at either the Carrington or Kererū dialysis centres, were between 18 and 80 years of age, tolerated toe pressure assessment, and were able to consent. Participants were excluded if TSBP could not be determined at baseline, revascularisation of both limbs had occurred within the past 3 months, they had undergone hallux amputation, or they had ulceration that would limit the ability to take a toe pressure measurement. Non-English speakers with no family/friends available for interpretation at the time of the dialysis session were also excluded. Participants were asked to refrain from having caffeine, smoking, or strenuous physical activity two hours prior to data collection, as per the technique paper by Tehan et al. [12].

### Recruitment protocol

Within the two centres, 108 patients were available for recruitment. After a four-week recruitment process, 30 people with ESRD agreed to participate in the pilot study. Recruitment occurred through a non-probability voluntary response sampling method, in which renal case managers identified potential participants based upon the inclusion criteria and then approached patients to determine their interest in participation. The names of potential participants were then passed onto the researcher. The researcher approached the patients during a dialysis session, discussed the protocol, consent processes, and participation date. The prospective participant could opt-out at this time or on the day of data collection. This sampling was deemed the most appropriate as the renal case managers have an in-depth knowledge of their clients and would be in the best position to approach those who may be interested; this recruitment method acknowledging that this can be a vulnerable population.

### Procedure

TSBP was measured bilaterally according to the protocol described by Tehan [12]. The protocol was modified with regard to the resting time before the initial TSBP measurement. Participants were rested in a 30 degree or lower supine position for 5 min prior to assessment, as opposed to the recommended 10 min. This protocol was adjusted to cause minimal disruption when participants were preparing for dialysis. Brachial systolic blood pressure was measured on one side only, which was determined by the presence of fistular, or by the participants' preference, using the dialysis machine. This procedure was performed before dialysis (T1), one hour after the start of dialysis (T2), and in the last 15 min of the dialysis session (T3). All TSBP readings were taken by R.C., a podiatrist with 18 years of clinical experience.

Demographic and medical history were collected by interviewing participants and reviewing medical records to obtain information on a history of ESRD, hypertension, dyslipidemia, previous stroke, previous heart attack, history of diabetes and history of diabetes-related foot complications, and smoking history. Dialysis notes were reviewed to determine type of dialysis used, duration of dialysis, interdialytic blood pressure variance, weight change, target weight, completion of a full dialysis session, and history of urination. Intermittent claudication was assessed using the Edinburgh Claudication Questionnaire [22]. Foot deformity was assessed using the 6-point scale, with one point assigned for small muscle wasting, hammer/claw toes, bony prominences, Charcot deformity and limited joint mobility [23]. A score of 3 and above indicates the presence of foot deformity [24]. Current callus was determined by the researcher and defined as minor, moderate or heavy. LOPS was defined by a 10 g monofilament assessment over the plantar hallux, first, and fifth metatarsal. If any of these points were absent, the participant was noted as having LOPS [7]. Frailty was self-assessed using two questions derived from the Clinical Frailty Scale [25]. Participants were asked “Do you go outdoors independently?” and “Do you exercise outside at all?”. If they were unable to go outdoors independently, they were scored 5 or above and were considered frail. If they do go outdoors independently, the self-assessed score was 1 to 4 depending on how often they exercise outdoors (1 = not frail, very fit and exercise often, 2 = not frail, fit, 3 = not frail and managing well, 4 = living with very mild frailty, but not dependent on others for daily help). The participant’s residential address was extracted from hospital notes and entered into the New Zealand Index of Deprivation [26], which is an area-based measure of socioeconomic deprivation in New Zealand and was derived from the 2018 census. This is an important indicator because of the relationship between socioeconomic status and mortality in New Zealand [27].

### Statistical analysis

Demographic and medical data were described separately for each group (diabetes, no diabetes), with n (%) used for categorical data and mean (SD) for continuous data. A linear mixed effects model was used to determine the variability in TSBP and TBPI across the three time points (T1, T2, T3) (primary aim) and whether this variability differed between people with and without diabetes (secondary aim). Time point (T1, T2, T3) was included as a within-subject fixed effect and participant group (people with diabetes, people without diabetes) was included as a between-subject fixed effect. The interaction effect (time point*participant group) was also examined. Repeated measures between right and left limbs were accounted for by the inclusion of a participant-specific random effect [28]. Mean estimates (adjusted for dependence between right and left limbs) were presented along with their 95% confidences intervals (CI).

A sub analysis assessing the difference in TSBP and TBPI variability between people with and without LOPS was also performed, due to the high number of participants with LOPS, to determine if this was a factor related to TSBP and TBPI. These analyses were also adjusted by participant group (people with diabetes, people without diabetes). All analyses were undertaken in IMB SPSS Statistics 25 with a *P* value of < 5% considered significant.

## Results

### Participant characteristics

Thirty participants were recruited, including 17 (57%) participants with diabetes and 13 (43%) participants with no diabetes. The median age for participants with diabetes was 56, (range 42–78) and 59 (range 24–79) for the no diabetes participants. Of the participants with diabetes, 10 (59%) were female, and of the no diabetes participants, seven (54%) were female **(**Table 1**)**. Socioeconomic deprivation as determined from the participant’s address revealed more participants with diabetes resided in areas of higher deprivation with 94% being in decile 5 or above, compared to 69% of the no diabetes participants within this study. Decile 1 represents the least deprived areas, decile 10 represents areas with the most deprivation.

**Table 1:** Participant characteristics

		PWD	No-diabetes	*P*-value
Sex (M:F)		10:7	6:7	
Age median (range)		56 (24-78)	59 (24-79)	0.62
Ethnicity, n (%)	Māori	2 (12)	1 (8)	0.72
	European	2 (12)	3 (23)	0.43
	Pacifica	11 (64)	7 (54)	0.56
	Indian	0 (0)	1 (8)	0.26
	Other	2 (12)	1 (8)	0.76
Decile of housing deprivation^a^ above 5, n (%)		16 (94)	9 (69)	0.07
Medical characteristics				
Type 2 diabetes, n (%)		13 (94)	0 (0)	< 0.0001
Diabetes duration, years, mean (SD)		22 (9)	0 (0)	< 0.0001
Hypertension, n (%)		13 (76)	9 (69)	0.42
Dyslipidaemia, n (%)		5 (29)	1 (8)	0.15
Smoker, n (%)		1 (6)	2 (15)	0.41
Previous/current heavy drinker, n (%)		2 (12)	1 (8)	0.72
Cerebrovascular diagnosis n (%)		0 (0)	1 (8)	0.85
Cardiovascular event, n (%)		2 (12)	2 (15)	0.78

*PWD* Persons with diabetes, *STEMI* ST-elevation myocardial infarction, *NSTEMI* Non-ST-elevation myocardial infarction, *n* number, *%* percentage

^a^Decile of housing deprivation is based on census information from 2018, decile 1 represents the area of lowest depravity, decile 10 represents the area of highest depravity

### Foot health characteristics

Foot deformity, minor callus formation, and peripheral neuropathy were more common in participants with diabetes than no diabetes. All participants reported low scores on the frailty grade **(**Table 2**)**.

**Table 2:** Foot health characteristics

		**PWD**	**No-diabetes**	***P*-value**
Foot deformity n (%)		7 (41)	2 (15)	0.14
Current callus	Minor, n (%)	14 (82)	9 (69)	0.41
	Moderate, n (%)	3 (18)	4 (31)	0.41
	Heavy, n (%)	0 (0)	0 (0)	
Loss of protective sensation, n (%)		9 (53)	6 (46)	0.49
Known PAD and known to vascular services (excluding fistular), n (%)		1 (6)	2 (15)	0.39
Revascularisation to lower limb performed, n (%)		0 (0)	0 (0)	
Intermittent claudication, n (%)		0 (0)	1 (8)	0.26
Frailty grade, mean (SD)		1.8 (1)	1.8 (0.9)	0.88

*PWD* Persons with diabetes, *PAD* Peripheral arterial disease, *n* number, *%* percentage, *SD* Standard deviation

### Haemodialysis characteristics

General haemodialysis characteristics are presented in Table 3. Persons with diabetes were on haemodialysis for a mean of 3.8 years and persons with no diabetes for a mean of 6.2 years. The aetiology of ESRD in participants with diabetes was attributed to diabetes in 88% of cases, and lupus and glomerulosclerosis to 6% of cases. In the no diabetes participants, the aetiology of ESRD was attributed to lupus (15% of cases), glomerulosclerosis (15%), hypertension (8%), glomerulonephritis (23%), uretic obstruction (8%), and was unknown in 30% of cases.

**Table 3:** Haemodialysis characteristics

	**PWD**	**No-diabetes**	***P*-value**
Haemodialysis duration, years, mean (SD)	3.8 (2.9)	6.2 (4.1)	0.07
Time on dialysis, hours, mean (SD)	4.8 (0.5)	4.7 (0.5)	0.61
Interdialytic weight change, kg, mean (SD)	1.99 (0.9)	2.04 (0.9)	0.91
Peritoneal dialysis before starting HD, n (%)	1 (6)	4 (30)	0.36
Interdialytic systolic blood pressure variation, mean (SD)	40 (21.5)	30 (16.3)	0.16

*PWD* Persons with diabetes, *HD* Haemodialysis, *n* number, *SD* Standard deviation, *kg* kilograms

### Primary aim: variability in TSBP and TBPI over time

Data showed a significant overall reduction in TSBP (*P* < 0.001) with all participants. There was a significant reduction in TSBP between T1 and T2 (mean difference [95% CI]: -8.34 [-14.22, -2.54], *P* = 0.006) and between T1 and T3 (mean difference [95% CI]: -11.95 [-17.83, -6.06], *P* < 0.001). No significant difference was found between T2 and T3 (mean difference [95% CI]: -3.61 [-9.49, 2.27], *P* = 0.17) **(**Table 4**).** There was no significant overall change in TBPI over time (*P* = 0.62).

**Table 4:** Mean toe pressure values and toe brachial pressure index values in PWD and no diabetes

		**All participants** mean (95% CI)^a^	**PWD** mean (95% CI)^a^	**No-diabetes** mean (95% CI)^a^
**Mean toe pressure (mmHg)**	**T1**	121.50 (105.72, 137.29)	126.85 (106.07, 147.64)	116.15 (92.39, 139.92)
	**T2**	113.17 (97.38, 128.95)	119.41 (98.63, 140.19)	106.92 (83.16, 130.69)
	**T3**	109.56 (93.77, 125.34)	111.88 (91.10, 132.67)	107.23 (83.47, 131.00)
**Mean toe brachial pressure index**	**T1**	0.79 (0.70, 0.87)	0.79 (0.68, 0.90)	0.78 (0.65, 0.91)
	**T2**	0.79 (0.70, 0.87)	0.80 (0.69, 0.91)	0.77 (0.64, 0.90)
	**T3**	0.80 (0.72, 0.89)	0.80 (0.69, 0.91)	0.81 (0.68, 0.94)

*PWD* Persons with diabetes, *T1* Pre dialysis measurement, *T2* Measurement at 1 h of dialysis, *T3* Measurement 15 min prior to conclusion of dialysis, ^a^Mean estimates adjusted for repeated measures on right and left feet (random effect)

### Secondary aim: difference in variability in TSBP and TBPI between diabetes and no diabetes participants

**Fig. 1:**
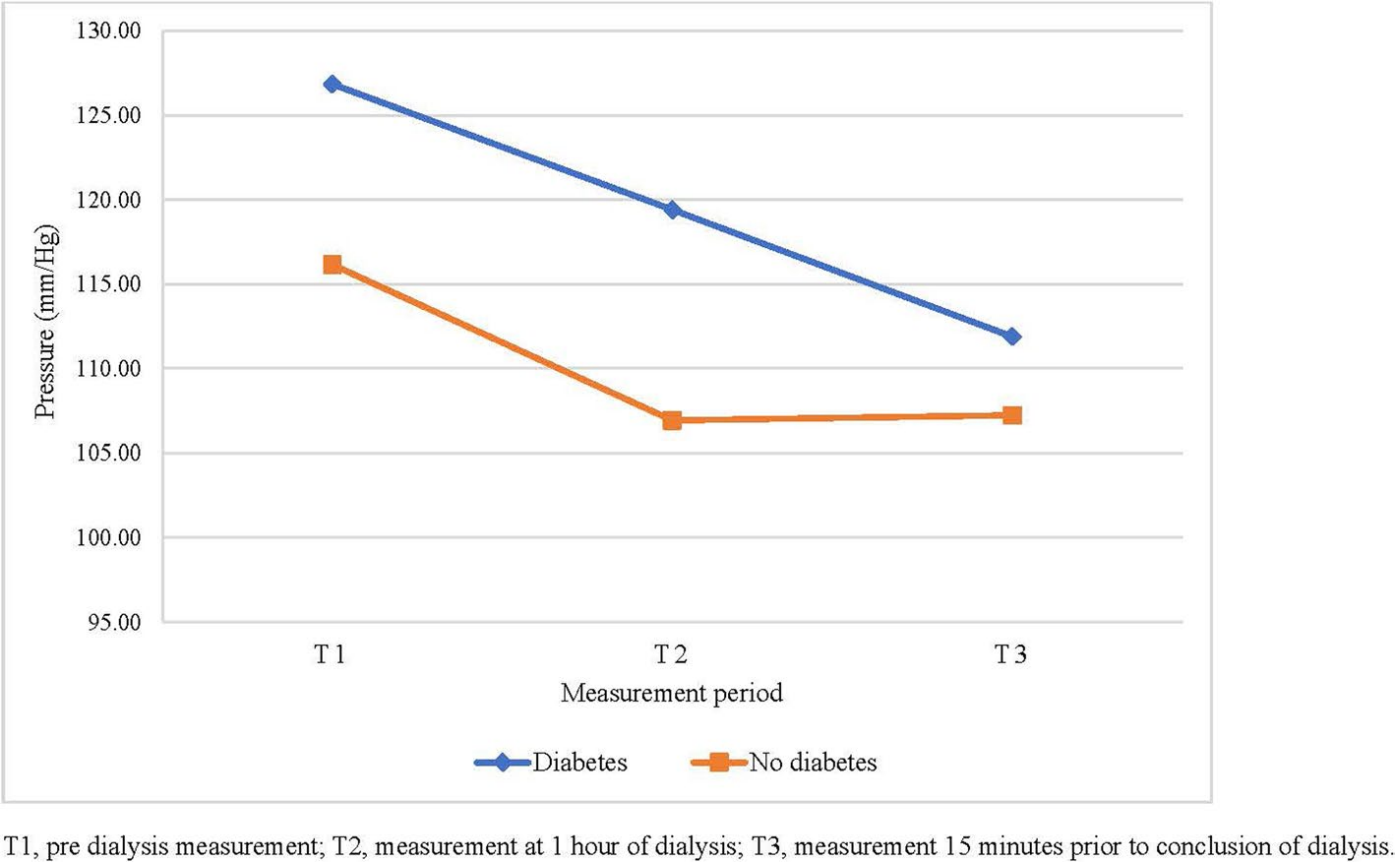
Mean toe pressure between T1, T2, and T3

**Fig. 2:**
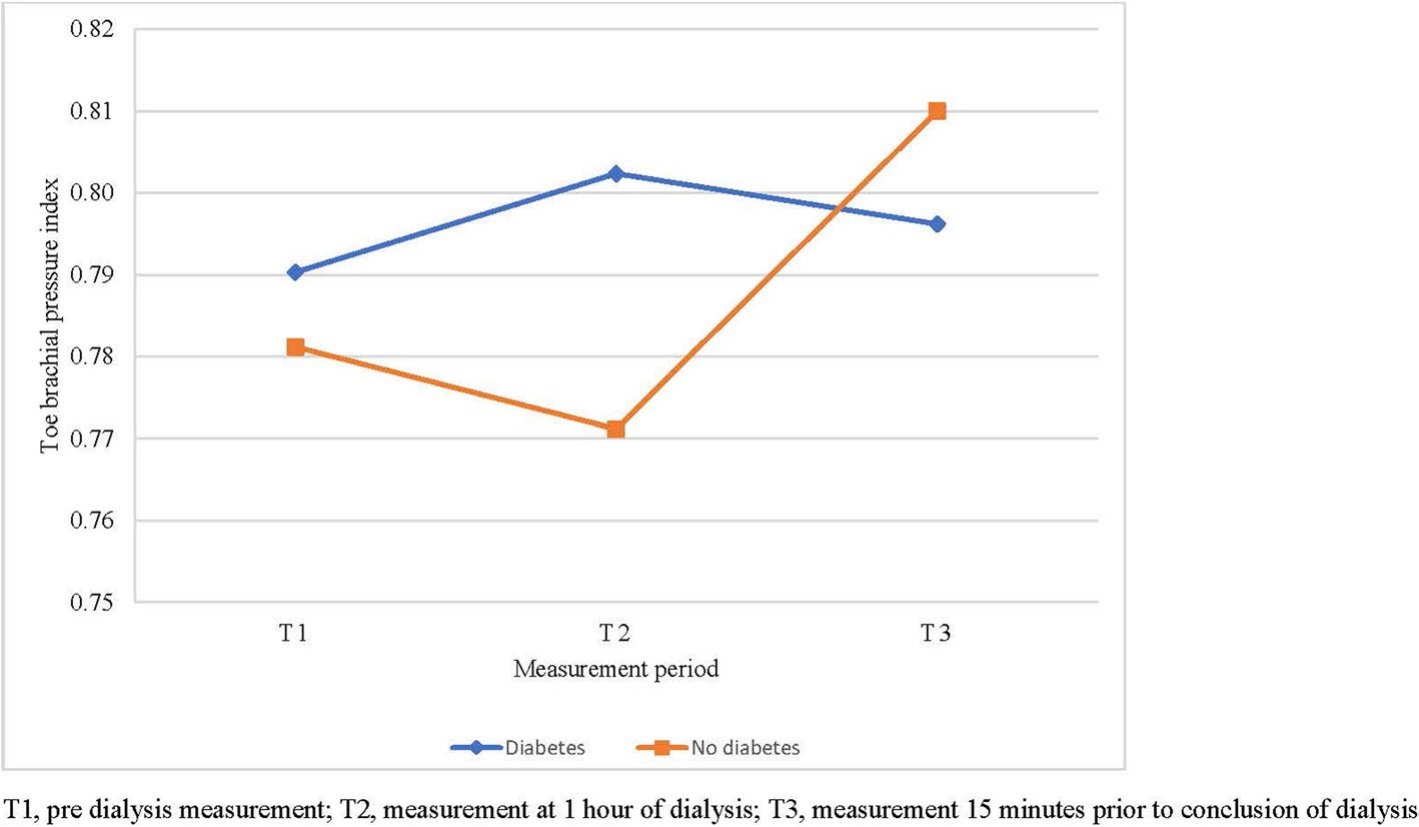
Mean toe brachial pressure index between T1, T2 and T3

There was no significant overall difference in TSBP between persons with diabetes and persons with no diabetes (mean difference [95% CI]: -9.28 [-40.20, 21.64], *P* = 0.54). There was no significant difference in TSBP across the three time points between people with and without diabetes (time point*participant group interaction) (*P* = 0.39) (Table 4). The change in mean TSBP is displayed in Fig. 1. There was also no significant overall difference in TBPI between people with diabetes and people without diabetes (mean difference [95% CI]: -0.01 [-0.17, 03.16], *P* = 0.91). There was no significant difference in TBPI across the three time points between persons with and without diabetes (*P* = 0.53) **(**Table 4**)**. The change in mean TBPI is displayed in Fig. 2.

### Sub-analysis: TSBP and TBPI in participants with and without loss of protective sensation

The sub-analysis included 15 participants with LOPS and 15 participants without LOPS. There was no overall difference in TSBP or TBPI between people with and without LOPS (*P* = 0.97, and *P* = 0.62, respectively) **(**Table 5**)**. There was no significant difference in TSBP variability across the three time points between people with and without LOPS (time point*neuropathy interaction) (*P* = 0.67), however, there was a significant difference in TBPI variability across these time points between people with and without LOPS (*P* = 0.003). There was no significant difference in TSBP or TBPI variability based on participant group (diabetes, no diabetes) between people with and LOPS (participant group*neuropathy interaction) (*P* = 0.32, and *P* = 0.84, respectively).

**Table 5:** Mean toe pressure and TBPI in participants with loss of protective sensation

		**No LOPS** mean (95% CI)^a^	**LOPS** mean (95% CI^a^
**Mean toe pressure (mmHg)**	**T1**	119.07 (96.86, 141.28)	122.67 (100.07, 145.27)
	**T2**	113.40 (91.19, 135.62)	111.90 (89.30, 134.50)
	**T3**	108.64 (86.43, 130.85)	108.40 (85.80, 131.00)
**Mean toe brachial pressure index**	**T1**	0.77 (0.65, 0.89)	0.80 (0.67, 0.92)
	**T2**	0.80 (0.68, 0.93)	0.77 (0.65, 0.89)
	**T3**	0.86 (0.73, 0.98)	0.75 (0.62, 0.87)

*LOPS* Loss of protective sensation, *T1* Pre dialysis measurement, *T2* Measurement at 1 h of dialysis, *T3* Measurement 15 min prior to conclusion of dialysis, ^a^Mean estimates adjusted for repeated measures on right and left feet (random effect), and participant group (diabetes vs. no diabetes)

## Discussion

This study presents New Zealand data related to the assessment of TSBP and TBPI during dialysis. Data showed that TSBP decreased significantly from baseline (T1) to the second (T2), and third (T3) TSBP measurements in both participants with diabetes and no diabetes. This is in contrast to the previous data indicating that TSBP was reduced only in persons with diabetes during dialysis and after dialysis [15]. Kay et al. postulated that the differences in TSBP between participants with and without diabetes may have been attributable to the presence of neuropathic sympathectomy [15]. However, there was no data provided indicating the prevalence of peripheral neuropathy in the study population.

TSBP decreased during dialysis, and this was reflected by a decrease in participants' brachial blood pressure during dialysis. The phenomenon of dialysis induced hypotension is thought to be related to the rapid shift in water from the intravascular compartment during haemodialysis, an impaired arginine vasopressin hormone regulation system (which influences optimal plasma osmolality function) and low vascular tone which can be present in people with ESRD [29]. Intradialytic hypotension is a common phenomenon during dialysis and was the most common cause of reduced dialysis sessions during the study. While TBPI can remain stable throughout dialysis, less information is available about the specific diagnostic limits of TBPI measurement. The current literature estimates that a TBPI < 0.7 could be diagnostic for PAD [30], with Høyer et al. suggesting < 0.64 and recommended more large-scale studies to define the diagnostic accuracy of the TBPI for PAD [31]. Despite these criticisms, TBPI has been shown to have higher diagnostic sensitivity compared to the ABI, particularly in the presence of medial arterial calcification, which is positively associated with ESRD [32].

The prevalence of a LOPS within the study cohort was lower than previously reported in people with ESRD. Jones et al. estimated upwards of 60% of people with ESRD have peripheral neuropathy [33]. Uremic neuropathy is a poorly understood side-effect of ESRD, thought to be related to uremic solutes, myoinositol and other molecules leading to a reduction of motor nerve conduction velocity [34]. It is difficult to differentiate between peripheral neuropathy and uremic neuropathy, and either neuropathy may have been present without loss of fine touch perception, which may have resulted in under reporting. Gold standard peripheral and uremic neuropathy assessment involves nerve conduction testing, which was not feasible for this study. LOPS testing through monofilament assessment is recommended in comprehensive foot examinations [35]. Additional neuropathy testing, such as biotheisiometer, tuning fork perception, and reflex testing may have increased the reported rates of LOPS in this study. Nerve conduction testing would have allowed for more precise peripheral and uremic neuropathy documentation.

In terms of comparing participants with and without LOPS, there were no significant differences in TSBP observed at the three time points. However, an interesting finding emerged regarding TBPI, as it consistently decreased throughout the course of haemodialysis in participants with LOPS. This finding was unexpected, considering the stability of TSBP across the time points. This result may be attributed to the variability within the small sample of participants with LOPS, which limits the generalisability of the findings. Nevertheless, this discovery emphasises the need for further large-scale investigation into the relationship between TBPI, LOPS, and dialysis.

The study findings should be considered with regard to some limitations. First, the participants were recruited from community dialysis centres, consequently people with ESRD dialysing in the hospital setting were excluded. Patients dialysing in the hospital setting may be considered medically more unwell than the participants recruited from our study centres. This may explain some of our outcomes, such as the cohort not being classified as frail, the low levels of current PAD, previous ulceration, amputations, and revascularisation present in participants. The hospital dialysis centres were not considered appropriate for data collection due to the tighter turn-around times between dialysing sessions, and space issues to conduct data collection. Second, the resting time prior to the T1 TSBP was reduced from the 10 min stated in the Tehan et al. protocol [12] to five minutes to reduce the time-burden on dialysis session times. As a result, this may have influenced the T1 result but was unavoidable given the tight time constraints surrounding dialysis sessions. TBPI has been shown to vary dependent upon rest time. Sadler et al. found a significant increase in TBPI when the premeasurement rest period was increased from 5 to 10 min [36]. Therefore, our initial T1 results may have been lower than expected, future studies should allow for greater resting time if possible.

Future work should consider comparison between participants receiving community-based dialysis and hospital-based dialysis. TSBP and TBPI analysis from participants with established PAD, previous amputations, and LOPS, would also provide more information on the appropriateness of obtaining these measures during haemodialysis. Additionally, longitudinal studies comparing results with ulceration, amputation, and revascularisation rates could assist in service planning within dialysis settings.

## Conclusion

Clinically, the results from this study should encourage use of the TBPI measurement on people whilst dialysing. TSBP reduced significantly throughout dialysis and therefore clinicians should be aware and take this into consideration. This reduction of TSBP during dialysis may have an impact on the healing capacity for people with active ulceration and may also be relevant in the development of lower limb complications.

## Data Availability

Data is available upon reasonable request.

## Abbreviations

ABI: Ankle brachial index
AUTEC: Auckland University of Technology Ethics Committee
ESRD: End stage renal disease
HDEC: Health and Disability Ethics Committee
LOPS: Loss of protective sensation
PAD: Peripheral arterial disease
TBPI: Toe brachial pressure index
TSBP: Toe systolic blood pressure
